# Efficacy of intralesional injection with PV-10 in combination with co-inhibitory blockade in a murine model of melanoma

**DOI:** 10.1186/2051-1426-2-S3-P120

**Published:** 2014-11-06

**Authors:** Shari Pilon-Thomas, Hao Liu, Krithika Kodumudi, Amy Weber, Ellen Moore, Amod A Sarnaik

**Affiliations:** 1 Moffitt Cancer Center, Tampa, FL, USA

## 

PV-10 is a 10% solution of Rose Bengal that is currently being examined as a novel cancer therapeutic. We have previously shown that intralesional (IL) injection of PV-10 into a single subcutaneous B16 melanoma tumor led to regression of both the injected tumor and uninjected B16 lung lesions. Tumor regression correlated with the induction of systemic anti-melanoma T cell immunity. In melanoma patients, IL injection of PV-10 has led to regression of treated lesions as well as untreated bystander lesions. In this study, we have examined whether IL PV-10 and co-inhibitory blockade could improve anti-tumor immunity and regression of melanoma. B16 cells were injected into C57BL/6 mice to establish one subcutaneous tumor. Treatment of this lesion with a single IL injection of PV-10 alone led to partial regression of the injected B16 lesion. Systemic administration of anti-CTLA-4 or anti-PD1 antibodies in combination with IL PV-10 resulted in increased tumor regression and improved survival in this model. Treatment with PV-10 also led to the induction of T cells that produced IFN-γ (495 ± 198 pg/ml) in response to B16 cells but not to irrelevant MC-38 cells. Combination therapy with IL PV-10 and anti-CTLA-4 led to increased IFN-γ responses to B16 (1235 ± 191 pg/ml, p < 0.05). In another experiment simulating heavy tumor burden using a bilateral model, systemic administration of anti-PD-L1 antibodies in combination with IL PV-10 led to regression of the injected B16 lesion as well as a bystander subcutaneous lesion on the opposite flank (p < 0.01 compared to mice treated with anti-PD-L1 antibodies or IL PV-10 alone). Together, these studies support the induction of increased tumor-specific immunity after co-inhibitory blockade in combination with IL PV-10 therapy.

